# Remarks on Serous Effusion within the Arachnoid Cavity

**Published:** 1850-04

**Authors:** Austin Flint

**Affiliations:** Prof. of the Principles and Practice of Medicine, and Clinical Medicine, in the University of Buffalo. Buffalo


// BUFFALO MEDICAL JOURNAL

AND

MONTHLY REVIEW.

VOL. 5. APRIL, 1850. NO. 11.

### ORIGINAL COMMUNICATIONS.

ART. I.—*Remarks on Serous Effusion within the Arachnoid Cavity; with Cases.* By AUSTIN FLINT, M. D., Prof. of the Principles and Practice of Medicine, and Clinical Medicine, in the University of Buffalo.

The phrase "Cavity of the Arachnoid" is used to express an imaginary space between the free surfaces of the arachnoid membrane. This membrane invests the superficies of the brain, and is reflected upon the inner surface of the dura mater, forming a shut sac, and, as in the case of any other serous membrane, the interior polished surfaces lying in contact, and unattached to each other. Strictly speaking, there exists no space or cavity here, more than in the instances of the pleura, peritoneum, etc. The arrangement, however, here, as in other serous structures, is such, that fluids may accumulate between the two free surfaces, or within the shut sac, in variable quantity, producing a condition which here, as in other situations, constitutes dropsy of the arachnoid. Dropsical collections thus situated, were formerly known with medical writers under the name of external hydrocephalus, the adjective *external* distinguishing this variety of encephalic dropsy from the variety, far more common, in which the effused fluid is contained within the ventricles of the brain. This distinction (which, if the views I shall present are correct, is a valid one,) has, of late, passed out of use, the occurrence of external dropsical effusions having nearly been lost sight of, or considered apochryphal, and the term dropsy of the brain, or head, has come to refer exclusively to accumulation of serosity within the ventricles. I believe I am correct in saying that the occurrence of serous effusion within the cavity of the arachnoid, either as a primitive disease, or as an element of other affections of the encephalon or other parts, is scarcely recognized by writers on pathology and practical medicine. If mentioned at all, it is only alluded to incidentally, and is not considered as a pathological occurrence of much importance. This remark is made with reference to standard, current authors, without professing to have made extensive bibliographical inquiries in order to ascertain if some one, or more, may not have sought to call the attention of the profession to the subject. The latter may be the case, but, if so, the present writer is unacquainted with the fact. It would certainly be expected that if a series of reliable facts bearing on this subject had been contributed, some notice of them would be found in the able and learned treatises on cerebral affections, which have latterly been published. Allusion is now made, more particularly, to the works of Solly and Burrows. In neither of these truly valuable works is the occurrence of dropsy of the arachnoid mentioned as an event of much pathological or practical importance.

During the past eight or ten years, several cases have fallen under my observation in which grave pathological phenomena have appeared to be fairly attributable to the presence of arachnoid effusion. These cases have seemed to demonstrate that apoplectic coma, and sudden death, may be occasionally due to this event, and that, occurring in the progress of other and various affections, it may, not very infrequently, prove the immediate cause of a fatal termination. Moreover, on analysis of these cases, in connection with pathological reasoning, it will, if I mistake not, appear, that, in many instances, a diagnosis may be made with considerable confidence.

To give an account of the cases referred to, and to submit such considerations relating to arachnoid effusion, as may therewith suggest themselves, are the objects of this communication.

A ew remarks farther, by way of introduction. Anatomically considered, there is a singular deficiency of information in standard works, respecting the normal condition of the arachnoid cavity. Resembling, as this membrane does, in structure, other serous membranes, and fulfilling, although within narrower limits, the same functions, we should expect that a certain amount of condensed halitus would be always found in the cavity after death. The reader, however, will consult, in vain, anatomical treatises for any precise statement with regard to this point. It is passed over very loosely. For example, Solly says, "the two internal surfaces of the arachnoid are closely in contact, and the bag which is between them is generally empty." Martin says, "a small quantity of serous halitus is always found interposed in the general cavity of the arachnoid." Wilson says, "the serous secretion of the true cavity of the arachnoid is very small in quantity as compared with the sub-arachnoidean fluid." Harrison says, "as in other serous membranes, the opposed surfaces of the arachnoid are always lubricated by a fine fluid or halitus, which facilitates their gliding motion, and obviates friction." The above quotations from these four authors, contain all that is said by them respecting the quantity of normal fluid to be found in the arachnoid cavity after death. The quotations show, in the first place, that there is a deficiency of exact knowledge on the point, and, in the second place, that the normal quantity, in the opinion of these anatomists, in a state of health, is quite small. This lack of precision is the more singular, because the fluid commonly known as the *cerbro-spinal fluid* has, of late, received much attention. The physiological importance of this fluid was particularly considered by Magendie, and considerable pathological consequence is attached to its variations in amount, by Burrows, and others. It is needless to remind the reader that the *cerebro-spinal fluid* is not contained within the arachnoid cavity. It is a sub-arachnoidean effusion, permeating the cellular tissue which connects the arachnoid with the pia mater, and filling the spaces formed by the extension of the arachnoid from one convolution to another without dipping between them.

Analogy leads to the presumption that the arachnoid cavity must be exposed to effusion of serum in morbid excess, since such is the case with all other serous structures. None is exempt from the liability to dropsical collections, and there is no apparent reason why the serous membrane of the encephalon should be an exception to the general rule.

But the inquiry cannot fail to arise, how is it that this has not been more fully and generally recognized as an important pathological event, liable to occur? That it has not is certainly surprising, but the fact may, in part, perhaps, be accounted for by the special care which is requisite in examination of the head after death, to determine the existence of arachnoid effusion, and its amount. In the mode ordinarily pursued of removing the brain from the skull, in autopsies, unless fluid exists in considerable quantity within the arachnoid cavity, it will be likely to be overlooked. It is, moreover, difficult to estimate the amount of effusion without particular pains have been taken to avoid the escape of blood from divided vessels, and of serum from the opened or ruptured ventricles, into the base of the cranium. Since practical writers do not speak of the presence of fluid in this situation as possessing notable pathological importance, close attention is not usually directed to this point in examinations, and when the quantity of fluid is so great as to arrest observation, it has not excited adequate interest. The precautions to be observed in order to ascertain the contents of the arachnoid cavity are, not to divide the dura mater with the head dependent, but, on the contrary, somewhat elevated; to remove the brain from the cranium before section of its substance, or without rupture of the investing arachnoid, allowing of the escape of the sub-arachnoidean fluid; and to remove it quickly after separating it from its connection with the medulla spinalis, observing, and endeavoring to estimate the quantity of blood and serum which escapes from the divided extremities. The best plan of removal of the brain, is to remove all above the tentorium before dividing the latter membrane, and afterward to remove the cerebellum. The fluid effused in the cavity of the arachnoid will be found at the base of the skull, and within the spinal canal. The quantity, in cases in which morbid effusion exists, will vary from half an ounce to six or more ounces. It may be translucent, like ordinary clear serum; more or less opaque from admixture of fibrin; or sanguinolent from hemorrhage, or the escape of blood from divided vessels in the examination. The practical importance of determining which of the latter explanations is applicable in particular cases, is too obvious to require remark.

The question may be submitted, in this connection, what would be expected to be the pathological or semeiological results of effusion into the arachnoid cavity. In answering this question, the situation of the fluid, relatively to the cranium and contents is to be considered. The portion of arachnoid below the tentorium would be most likely to furnish a morbid effusion. The arachnoid at the base of the brain is thicker than over the convex surfaces of the cerebrum. It is more apt to be the seat of subacute inflammation, and tubercular deposits are generally confined to this locality. But wheresoever the effusion takes place, it will occupy, by gravitation, first the spinal canal. Here the accumulation would not occasion serious trouble until the quantity was sufficient to occasion compression of the medulla oblongata. Now, what are the functions, and what the physiological importance of the medulla oblongata? Physiologists are agreed that, in addition to its motor and sensory tracts, this portion of the cerebro spinal system contains the ganglionary centres presiding over the instinctive sense and acts of *respiration* and *deglutition*. Hence, the phenomena denoting compression from the presence of fluid at the base of the brain, should be manifested in the functions just named. The important bearing of this fact on diagnosis is at once apparent. It may be reasonably conceived that if either, or both these functions manifest symptoms of disorder, which may be determined to proceed from a cause within the encephalon, and, at the same time, the intellectual faculties are not disordered, we have good grounds for the inference that the medulla oblongata is particularly involved in the disease. It will be seen how far facts are consistent with this theoretical view. It is needless to say, that no portion of the cerebro spinal axis is so directly concerned in the continuance of vital functions as this. Experiments have demonstrated that the entire brain above the medulla may be sliced away, in inferior animals, without death immediately following, and that destruction of the entire spinal cord below, is not incompatible with life for a short period. But to suspend the functions of the medulla oblongata (such is its connection with respiration, and such the connection of other vital functions with the latter) is to destroy life instantaneously. It is easy thus to understand why compression of the medulla from the rapid effusion of fluid may occasion sudden death.

The train of symptoms attending the extravasation of blood at the base of the brain, (an event of rare occurrence,) must, it may be presumed, be the same as when the same amount of compression is produced by an accumulation of serum in the same situation. The difference in the character of the fluid, in the two instances, cannot be supposed to make any material difference in the phenomena which are in both due to mechanical compression. Hence, cases of extravasation of the base of the brain may be cited, as illustrating the results which should be expected in cases of serous effusion. Histories of cases of extravasation, can, doubtless, be found, by consulting works which treat of cerebral diseases. The following case has been casually met with within a few days. It is taken from the late re-published English treatise on the diseases of children, by Charles West, M. D. In copying the case, I have italicised the symptoms to which the reader's attention is particularly directed, as the most prominent symptoms belonging to the history, aside from the convulsions. I should state that the author does not appear to attach any special import to the symptoms italicised. He does not allude to them, as, in any way, possessing diagnostic value, denoting the particular seat of the compressing cause. It will be observed that the extravasation was not exclusively limited to the brain, at the posterior part, and, therefore, the symptomatic phenomena were less distinct and significant than they might otherwise have been.

"Some years ago, I saw a little boy, five weeks old, the child of healthy parents, and who had been perfectly well for the first fortnight after his birth; he then, without any evident cause, grew drowsy, and vomited often, and his skin became quite jaundiced. His abdomen at this time was large and hard, and he cried when pressure was made on the right hypochondrium, and these symptoms still continued when he was brought to me. A leech, now applied on the right side, drew a good deal of blood, and the hæmorrhage was stopped with difficulty; the bowels, previously constipated, were acted on by small doses of calomel and castor oil, and in three days the child lost the yellow tinge of his skin, became cheerful, and seemed much better. He was now, however, on the 18th of July, *suddenly siezed with hurried respiration*, and great depression, soon followed by violent convulsions, during which he screamed aloud. At the same time it was observed that his left hand had begun to swell, and to put on a livid hue; and, on the 20th, the right hand also became œdematous. His whole surface grew quite sallow, and on the day before he died, the œdema of the left hand had much increased; the liver had become considerably deeper, and there were small spots of extravasated blood over each knuckle. The right elbow was slightly livid; the right hand much swollen, but of its natural color; and a small black spot had appeared under the chin, corresponding to the knot of the cap-string. The fits recurred very frequently, the child, in the intervals, lying quite still; the pupils were contracted, and the condition seemed to be one of extreme exhaustion rather than of coma. *On the 20th, the power of deglutition was lost*, and after several returns of less violent convulsions, the child died at 9, A. M., July 21st; about sixty hours after the occurrence of the first fit.

"The sinuses of the brain were full of fluid blood; a black coagulum, three or four lines thick, covered the whole posterior part of both hemispheres, extending from the posterior third of the parietal bones, occupying the whole concha of the occipital bone, and reaching along the base of the skull to the foramen magnum. A little blood was likewise effused about the anterior part of the base of the brain, though the quantity was very small in comparison with what was found at the posterior part. The substance of the brain was very pale, and all the organs of the body were anæmic, except the liver, which was gorged with fluid blood, while the heart was quite empty. The ductus arteriosus was closed, the foramen ovale admitted a probe with ease, the ductus venosus admitted one with difficulty."

The following is from Abercrombie on diseases of the brain, [Am. Ed. p. 141]:—"A man, mentioned by Dr. Douglass, had been for three months affected with pain in the forehead, which generally obliged him to sit with his head leaning forward; he had bad appetite, and disturbed sleep, but no other symptoms. *He died suddenly in an attack resembling syncope*, having been for a day much better, with good appetite, and quiet sleep. An encysted abscess was found in the middle of the cerebellum, *and a rupture of the left lateral sinus, which probably was the immediate cause of death.*" [Italics by the writer of this article.

The following cases were communicated by D. J. T. Francis, M. B., for Guy's Hospital Reports, (Eng.,) several years since, together with other cases, illustrating some of the forms of sudden death:—

"A man, æt. 56, who, although he had been disabled for four or five years, by a partial palsy of the left arm and leg, continued in good general health. One day, while standing up in the middle of the yard, passing his urine, (he had considerable difficulty in micturition,) he was observed to glide easily to the ground; he gave two or three short cries, but there was no struggling. Within two minutes after he fell, being close at hand, I saw him; but from this time there was no attempt at inspiration, the whole apparatus being quite motionless, although the heart continued to beat, pretty forcibly at first, and afterwards with a series of three or four faint pulsations, and gradually lengthening intervals, for nearly three minutes after I first saw him. The face was not remarkably livid; the pupils were somewhat, but not much, dilated. From these circumstances, an effusion of blood interfering with the respiratory portion of the medulla oblongata and annihilating the perception of a 'besoin de respirer,' was predicted."

*Dissection.*—Extensive effusion of blood at the base of the brain and around the medulla oblongata. Lateral ventricles empty; a few clots in the fourth ventricle. The arteries at the base were studded with opaque atheromatous spots; on gently throwing a current of water into the basilar artery, two jets issued from apertures in the posterior cerebral arteries. *Heart* sound, or nearly so. Some traces of an old pleuritic and pneumonic attack in the left lung. Abdominal viscera sound.

*Case* 2.—A man, æt. 58, having been absent for a minute in the water closet, was heard to fall on the ground. He was found comatose; the breathing was slow and heavy; the countenance was of a sublivid hue; the pulsations at the heart and wrist were full and powerful, and continued most distinctly for some time after all attempts at respiration had ceased. The pupils were at first contracted; and afterwards somewhat, but not greatly, dilated. Death took place in about eight minutes. This man had had an attack of partial hemiplegia six years before.

*Dissection.*—The lateral ventricles of the *Brain* contained a large quantity of bloody serum and florid clot. Great softening of the *fornix* and of the ventricular parietes. There was extensive effusion of recent coagulated blood at the base of the brain. The fourth ventricle was filled with a coagulum, which, after firmly investing the medulla oblongata, passed down the spinal canal, as far as it could be seen. There was no appearance of disease in the posterior cerebral or basilar arteries; but, when water was thrown into the latter, it escaped freely from the vessels in the fissures of Silvius, which were becoming opaque and hardened. *Lungs* almost entirely healthy. Pericardium adherent in nearly its whole extent. Left ventricle of the *heart* much thickened; valves sound. Both kidneys granular.

*Case* 3.—A man, æt. 54, a tailor, soon after eating a hearty breakfast of porridge and milk, and while sitting in the usual cross-legged posture at his work, suddenly complained of pain in the head, and fell from his seat, becoming almost immediately insensible. He had returned from the water-closet only a few minutes previously. He died within five minutes from the moment of the seizure.

*Dissection.*—All the sinuses of the *brain* were enormously distended; its substance throughout seemed healthy. Ventricles empty. No appearance of extravasation any where. After the brain was removed, several ounces of blood flowed from the spinal canal and the base. Both *lungs* were excessively gorged with fluid blood. The *heart* was small, pale, and flabby; left ventricle thin and lacerable; when cut into, there escaped about four ounces of dark fluid blood, much of which came from the aorta. The right side was not quite so full of blood; the ventricle containing a loose clot, the only one found in the body. The abdominal viscera were dark and congested, but seemingly healthy otherwise.

In the following remarks by the reporter of the above cases, it will be observed that the explanation of the mode of death is the same which will be applied to cases presently to be given. The author calls attention to an interesting peculiarity appertaining to the action of the heart, which will also be noted in some of the cases which are to follow:—

" With respect to these three, and such like cases, we may presume that the cause of death is pressure upon the *medulla oblongata*—whether applied immediately to the part from extravasation, or indirectly, from the general distension of the cerebral mass with blood—and the consequent suspension of the function of Respiration.

" Independently of the pathological interest which attaches to these cases, they were alike characterized by a remarkable symptom, which, as it was carefully observed, could not fail to arrest the attention;—I mean the decided manner in which the action of the heart and the pulse at the wrist were prolonged, after the respiration had completely ceased. This circumstance was obviously due for its cause to the especial manner in which the hæmorrhage that had taken place had compressed the medulla oblongata, and so annihilated the function of the part which presides ove the respiratory movements; whilst the heart, being less completely under the influence of that portion of the nervous system, had continued in its action, ceasing only from the obstructed circulation through the lungs. Experiment and previous pathological observation had long since established the fact of the dependence of the respiration upon integrity of the medulla oblongata; but the availability of the priority in cessation of the action of the heart or lungs, as a means of elucidating the causes of sudden death, appeared further to be an object well worthy of careful investigation."

With these preliminary considerations, I proceed to present a series of cases, bearing on the subject, submitting, in connection with each case, such remarks as are suggested by its peculiar features.

Case 1.—*Sudden attack of convulsions, and death in* 18 *hours. Moderate effusion within Arachnoid Cavity. Peyerian glands, and the mesenteric glands enlarged.*

I was summoned to visit H. T., aged 4 years, on the 20th of April, 1842 She had been attacked with convulsions shortly before, which continued for a few moments, and had ceased before my arrival. The convulsions consisted of rapid movements of the upper extremities, followed by tetanic rigidity. I found her with *short and hurried respiration*, and disposed to sleep. Pulse was rapid and small. Considerable lividity of extremities was afterward observed, with coldness. On application of heat, immersing hands and feet in warm water, reaction appeared to occur, and the face became flushed. I learned that on the 18th inst., she had seemed rather unwell, and at night vomited several times. She was also restless, and had febrile movement.

On the 20th inst., before the convulsions occurred, some appearance of an eruption on the feet and hands had been observed, Some soreness of throat had also been complained of. These circumstances had led the parents to apprehend scarlatina, with reference to which, some saffron tea had been administered. She had taken egg for breakfast. The abdomen felt voluminous, but not hard, nor much distended.

I directed an emetic of Ipecac, grs. xv., with calomel, grs. vi., which discharged from the stomach the food taken at breakfast, unchanged.

About an hour after the first attack of convulsions, a second occurred, in my absence, The latter was light. I directed, after the second attack calomel, grs. xv., followed by S. Magnesiæ, ℥ss, with Nit, Potassæ, grs. xv. Sinapisms were applied to the epigastrium, spine, and calves of the legs. The head was kept wetted with spirit and water, and several enemas of salt and water were given.

The patient remained through the forenoon without return of convulsions. She was disposed to sleep, which was profound, *and with somewhat stertorous respiration,* but she was roused without much difficulty. When roused, her attention was engaged with toys. She sat up, some of the time, playing with dolls, etc.

In the afternoon, the tendency to sopor increased, and, at length, eventuated in complete coma. Pulse continued rapid, skin hot, without the lividity observed in the morning; face flushed.

At 7, P. M., four leeches were applied to the temples, which were followed by free bleeding, reducing the heat of the skin, and occasioning some nausea and gaping. The stupor, however, was not relieved, *which was accompanied by much acceleration of, and noisy respiration.*

At midnight I was summoned, and found pulse scarcely perceptible, extremities cold, complete coma, *very short and quick respirations, accompanied by tracheal rattle, difficulty of deglutition,* great restlessness. Death occurred at 2, A. M.

*Examination* 12 *hours after death.*—Present, Drs. Charles Winne, and Alden S. Sprague.

Adipose substance abundant, dark discoloration of posterior part of neck and shoulders from gravitation of blood.

*Head.*—Adhesions of dura mater to skull tolerably firm. Vessels of pia mater moderately injected. No opacity of arachnoid. Section of cerebrum displayed some dark points. Lateral ventricles contained no fluid beyond moisture. Plexus choroides deeply injected. Sinuses moderately filled. At base of skull, after removing the brain, about half an ounce, as estimated, of serum appeared, and about the same quantity in addition escaped from the spinal canal. A portion of the medulla spinalis, about an inch from the oblongata, was removed and examined, exhibiting nothing unusual.

*Chest.*—No pleuritic adhesions. Lungs engorged at dependent portion, otherwise perfectly normal. Thymus gland not enlarged. Heart normal.

*Abdomen.*—Several of the Peyerian patches (about 12) thickened, of dark red color. No ulcerations. Solitary glands strikingly developed. Mesenteric glands hypertrophied, several as large as filberts. Those nearest the cœlum most enlarged. Small intestines quite empty. One lumbricoid observed. Stomach, cœlum, and colon not examined.

The following circumstances belonging to the history of this case should not be omitted. She had suffered from diarrhœa about one year prior to her decease. She then became much reduced, but afterwards recovered her usual flesh and strength. She had had an attack of vomiting a few weeks before her death, accompanied with febrile symptoms, which continued for several days. From the latter she had not seemed entirely to have recovered, up to the time of the fatal attack.

*Remarks.*—Some of the circumstances in the history of this case, go to sustain the hypothesis that the disease was scarlatina. At the onset of that disease, as is well known, convulsions and coma, leading to a speedily fatal result, sometimes occur. Whether this be considered the most probable view of the case or not, the most rational inference from the history during life, and the morbid appearances after death, in the opinion of the writer, is, that the occurrence of the cerebral symptoms, and the issue of the case, were due to the sudden effusion of serum into the cavity of the arachnoid, preceded and accompanied, probably, with cerebral congestion, the latter standing to the effusion in the relation of causation. May not this be the true explanation in some of the cases of scarlatina which prove rapidly fatal in the access of the disease?

The presence of the intestinal lesions characteristic of typhoid fever, is an interesting feature of the case, which it is foreign to my present purpose to discuss.

Case 2.—*Miscarriage, followed by profuse and alarming flooding. Subsequent dyspnœa, occurring in paroxysms, which ceased, and were succeeded by vomiting. Suddenly loss of consciousnes, Respiration much affected, and death in a few hours. No examination after death.*

The history of this case is given here, because it comes next in chronological order among those marked for reporting in this article. It is introduced to show, from the symptoms, the probability that the sudden supervention of the cerebral phenomena, and the fatal termination, were due to effusion within the anachroid cavity.

The patient, a married lady, aged 36, had had several children, and frequent miscarriages. She aborted in April, 1842, being about two months advanced in pregnancy. Considerable flooding attended the abortion. A few hours afterward, profuse hæmorrhage occurred in my absence, and before it could be arrested by the tampon and other measures, so great a quantity of blood had been lost, that alarming syncope ensued, and she had well nigh died at that time.

In a few days subsequently to the above occurrence, she began to suffer from paroxysms of dyspnoea, resembling asthma, occurring at night. These paroxysms gradually abated, and in eight or ten days had entirely ceased. They were considered and treated as neuropathic in their charaeter. As the paroxysms of dyspnoea abated, vomiting became a troublesome symptom The acts of vomiting recurred several times during the day, and were evidently paroxysmal, not excited by ingesta, unattended by nausea, in the intervals, and excited by any thing producing a strong nervous impression, for example, the sudden entrance of a person into her room. This state of things continued for several days. The pulmonary symptoms had entirely ceased. The alvine dejections were not unnatural. Pulse uniformly accelerated, and vibratory. Much prostration.

On the morning of the 25th of April, I was called to visit her at 2 o'clock. She had been very restless during the night, and complained of seeing disagreeable objects. Occasional retching as usual, and during apparent sleep, constant moaning. On being asked where she felt distress she replied, "all over." I left her, after administering morphia, gr. 1-8, apparently more comfortable, at 5, A. M. At 6½ I was again summoned, the symptoms being the same. I directed repetition of the morphia. At 7, A. M., she appeared to fall into a quiet sleep. At 8, I was summoned in haste, and found *respiration catching, the alæ nasi much contracted with each inspiration*, extremities cold, aspect hippocratic, *The pulse, however, remained considerably developed*, (frequency not noted.) *She had complained, for the first time, of acute pain in the head.*

Stimulants were exhibited, friction and heat to extremities, sinapisms to nucha. With these measures warmth of the surface was restored, but the respiration continued catching, in paroxysms, followed by intervals of profound somnolency, in which the respiration was tranquil. She soon lost all consciousness, which did not return, and death occurred at 9, P. M. Up to a few moments of her death, the skin remained warm, and the pulse continued developed. *She continued to have frequent paroxysms of catching respiration, and suspension of respiration for several seconds, followed by deep and quiet somnolency. These paroxysms were so severe that dissolution was expected each time that they occurred. Deglutition became impaired, and lost toward the close of life.* Tracheal rattles commenced about an hour before death.

She opened her eyes occasionally, until two hours before her death, but giving no evidence of recognizing persons about her. The eyes had a striking brilliancy, not the filmy appearance generally noticed before dissolution. Copious perspiration occurred about twenty minutes before death.

*Remarks.*—This would seem to be a case in which, from the history, the most rational mode of accounting for the last symptoms, and death, is to suppose effusion into the arachnoid cavity to have occurred. The development of somnolency, proceeding rapidly to complete stupor, attended at the outset, but not preceded by pain in the head, or other cerebral symptoms, characterised by striking disorder of the respiration, the pulse remaining developed, the patient evidently dying of apnoea—these are the points, taken in connection with the absence of symptoms denoting extravasation into the cerebral substance, (e. g. Paralysis,) upon which that conclusion was based. The arachnoid effusion was an intercurrent pathological event, a species of accident, occurring in the progress of other disorders. It was probably in this, as in most other cases, dependent on cerebral congestion. It may be said that the cause of death in this, as in other similar cases, is congestion of brain, but congestion alone, would not occasion death in so short a space of time, nor would the evidences of compression of the medulla oblongata be so prominent from the commencement of the cerebral attack.

Dr. R. C. Wood, Surgeon, U. S. Army, attended in consultation in this case.

Case 3.—*Chronic Arachnitis, without the presence of symptoms denoting cerebral disease. Sudden development of apoplectic symptoms, terminating fatally in a few hours. Examination after death. Exudation of firbin and serum beneath arachnoid; effusion of turbid serum at the base of brain. Serous apoplexy.*

I was invited by Dr. Wilcox, of this city, to attend a post mortem examination on the 27th September, 1845. The following account of the case was furnished by Dr. W.:

The night previous (26th), at 10 o'clock, he was requested to visit the patient. An unusual noise had attracted attention to his room (at a hotel,) where he was found lying on the floor in a state of insensibility. The extremities being cold, some brandy was at once administered, and frictions employed until Dr. Wilcox arrived. Dr. W. found the pulse 120, small and hard, face flushed; *respiration at times slow and stertorous, constantly deep and heavy, but occasionally labored and noisy.* Hands carried up to face, and one up to head, fingers partially clenched, with occasional convulsive movements of them. Extremities warm.

He first poured water from a pitcher on to the head for several minutes, and afterward applied napkins wetted in cold water. Half an hour after Dr. W. arrived, the patient began to gulp, as if disposed to vomit. This led Dr. W. to prescribe an emetic of antimony. The antimony did not occasion free vomiting: it caused him to eject, by several acts of gulping, first, a fluid, the appearance of which was not observed; next, indigested food, and lastly a yellowish fluid. The pulse became more developed, and the doctor then bled the patient ℥xxx. The blood flowed freely and with force. He noticed, in addition to the other symptoms, that the right pupil was considerably dilated, and the left of ordinary size. and both immovable on the approach of light.

The immediate effects of the bleeding were, reduction of the volume of the pulse, less laborious and noisy respiration, general muscular relaxation. Shortly after the bleeding, the doctor noticed that the left side of the face appeared to be paralyzed.

*The respiration soon became accelerated, and more laborious than before,* accompanied by the tracheal rattle, and death occurred at 10 A. M., the following morning.

It appeared that the patient had recently come to this city. He was about 32 years of age. His habits were dissipated. He had had syphilis. For two or three weeks after his arrival here, he had drank to excess, being intoxicated much of the time. For the week previous to the fatal attack, he had indulged to much less extent. For a week or so before his death, he had been under the care of Dr. Bailey, of this city. He complained of chills and debility. His aspect was morbid, *but he never complained of pain in the head, nor were there any prominent symptoms leading to the suspicion that the brain was the seat of disease.* He was able to be about, and was walking out of doors the day before his death.

*Post Mortem Examination.*—Present, Drs. White, Hamilton, Bailey, Mixer, Wilcox, and a physician from Michigan, name unknown. The examination was made five hours after death, and was necessarily limited to the head.

On removing the calvarium, and detaching the dura mater, the entire surface of the brain presented an opaque appearance. The opacity and elevation of the arachnoid were sufficient to conceal the convolutions. Turbid serum exuded in small quantity at whatever point the arachnoid was divided. The arachnoid and pia mater were readily detached, and when examined on the inferior surface, seemed coated with a layer of fibrin. The pia mater seemed lost in the fibrinous coating. The cerebral substance was of normal consistence. The incised surfaces of the brain presented a few red points. At the base of the brain, the arachnoid opacity was slight. At a few points, however, on the cerebellum, fibrin was effused beneath the arachnoid. *Considerable effusion of turbid serum existed at the base of the brain—the quantity was estimated to be between three or four ounces.* In one of the lateral ventricles about half an ounce of turbid serum existed. In the other ventricle a slight quantity.

*Remarks.*—This case is interesting, as affording an instance of considerable and extensive arachnitis, which had doubtless existed for some time, without any symptoms being present indicating the head to be the seat of disease prior to the apoplectic seizure.

But the point in the history which is chiefly important with reference to the subject under consideration, is the apoplectic seizure. To what pathological condition in the encephalon, suddenly induced, was this attributable?

The sero-fibrinous effusion beneath the arachnoid was probably not a new event, but had existed for some time; nor is it probable that a sudden increase of the effusion in this situation would have produced the violence of the symptoms characterizing the fatal attack, and its speedy fatality. The quantity of effusion into the arachnoid cavity was large, and is it not the most rational view to suppose, that an increase in this effusion induced the sudden coma, and, by compression of the medulla oblongata, occasioned death by apnoea.

It is not improbable that the sub-arachnold effusion, and the effusion into the lateral ventricles, may have taken place, or become increased, simultaneously with the increase of effusion into the cavity of the arachnoid. The large amount of fluid in one of the lateral ventricles may perhaps explain the facial paralysis. This case is entitled to be considered one of *serous apoplexy*.

Case 4.—*External Hydrocephalus.*—October 11th, 1845, I attended, by invitation of Dr. Treat, the examination of the head of a child eighteen months old.

Dr. Treat furnished the following history of the case:

"Saw child of Gustavus Yost, aged eight months. Learned that the mother had had previous miscarriages, and that this was the first child born at full period of gestation. The child was apparently healthy, until the second month of its birth, when attention was called to the distended blue veins of the forehead, and the glossy appearance of the head. Soon afterward the head became obviously enlarged. Child had been visited by Dr. Wood and Dr. Long; U. S. Garrison, and by two or more German physicians. The child lay supine, the face in the depressions of the nose, at the orbit, and under lids, was quite red with erysipelatous inflammation. Head preternaturally large, and sutures open. Diarrhœa, with dark, mucous, offensive stools. Pulse depressed, neither quick nor frequent. Intellect languid, not easily aroused, save by elevating the head. Not disturbed by light or noise. Moans constantly. Stomach regurgitates food.

Child died several days after this visit by Dr. T. The subsequent symptoms not ascertained.

*Examination of head nine and a half hours after death.*—The calvarium and brain were removed, the subject lying on the face. The dura mater being wounded, considerable fluid escaped before the brain was denuded. Several ounces of bloody fluid (estimated four ounces), remained at the dependent portion of skull, the concavity of the frontal bone. Ventricles empty. No fluid at base of brain. Nothing abnormal discovered in the condition of the brain or its meninges.

*Remarks.*—This case is simply given as a case of external hydrocephalus. The portion of the cadaver accounts for the situation of the fluid within the arachnoid cavity. The symptoms preceding death, if ascertained at the time, were not recorded, hence the case has no practical bearing on the present subject. May not position of the body be available, to some extent, as a therapeutical measure in, cases in which effusion is supposed to have taken place within the arachnoid cavity: in other words, may not the medulla oblongata be relieved of direct compression, by position on the face? It would seem that this case offered a favorable opportunity for paracentesis, if that operation be ever allowable in encephalic dropsy.

CASE 5.—*Persistent Vomiting. Disease of head not suspected. Effusion into arachnoid cavity, and beneath arachnoid, without other evidence of cerebral disease. Cirrhosis.*

The patient, in this case, was an inmate of the alms-house in this county. She was a female prostitute, about 30 years of age, of very dissipated habits. and had been several times an inmate at the alms-house with syphilis. She was received about four weeks before her death. Her ailment was the constant rejection of every thing ingested, and this had been the case for two weeks before her admission. She was treated with calomel and opium, blisters to the epigastrium, and spine, laudanum injections, and beef tea by injection. Up to two days before her death, her intellect remained unaffected. She then began to wander, and for twenty-four hours before her death she was in a comatose state. She never complained of pain in her head. I did not see the patient during her life. The particular symptoms toward the close of life, much to my regret, are not recorded.

*Post Mortem Examination.*—*Head.*—Considerable effusion beneath the arachnoid universally. In dividing the dura mater, considerable fluid escaped from the arachnoid cavity, and remained upon the tentorium. At the base of the skull about three ounces, as estimated, of fluid was observed, and on elevating the body, from half an ounce to an ounce escaped from the spinal canal. The fluid was opaque. Very slight effusion into the ventricles. The arachnoid appeared somewhat firmer than usual. Vessels of pia mater but slightly injected. Brain pallid, and free from any morbid appearances.

*Stomach* contained some six or seven ounces of greenish colored fluid. The mucous membrane presented some punctated redness, not extensive nor considerable, otherwise normal. Superficial appearance of intestines presented nothing unnatural.

*Liver.*—Much enlarged, extending into left hypochondrium, elevating the heart above its normal position. The structure of the liver presented marked granular degeneration, or cirrhosis.

*Remarks.*—This examination was made with reference to an anatomical object, hence it was not prosecuted extensively, or minutely enough, for a complete pathological investigation of the case. Nor is the history sufficiently full for the latter purpose. Not appreciating the importance of effusion into the arachnoid cavity at the time, I did not take pains to obtain a detailed account of the symptoms after head trouble became apparent, so as to establish a relation between them and the existence of the effusion. With its imperfectness, as respects details, the case is only interesting from the mere fact of the arachnoid effusion, and from the disease having been referred exclusively to the stomach, in consequence of the persistent functional disturbance of that organ. It is not easy to decide at what period of the disease the cerebral effusion occurred, or whether this effusion was primary, or secondary, as regards the disorder of stomach. That the effusion was directly concerned in the fatal termination, seems to me probable, although it is less demonstrable, in this instance, than in some of the others given, owing to want of information respecting the mode of death, or the symptoms preceding that event.

CASE 6.—*Apoplexy, without premonition, proving fatal in a few moments. Intense cerebral congestion, and effusion into arachnoid cavity.*

Mr. B., aged 79, had generally enjoyed robust health; been subject, especially during the preceding summer, to attacks of pain in the epigastrium, extending to the left upper extremity, running down to the fingers (angina pectoris); occasionally troubled with dyspnœa on exercise, but not uniformly; never complained of palpitation. He was in the habit of exercising actively, and was accustomed to indulge freely in eating, without indigestion, or other inconvenience. He retired to bed, Oct. 13th, 1845, at 9½ o'clock, after spending the evening at home, as well as usual, engaged in cheerful conversation. Had eaten, as usual, heartily at supper, at 6

o'clock. Had made no complaint of any ailment, either on that day, or for some time previously.

At 10½ his wife was awakened by an *unusual character of respiration*, and found him insensible. She called her son, who resided in the house, and had not retired. On his reaching the bedroom, his father was gasping at lengthened intervals. He gasped but a few times afterwards, and expired. There were no convulsive movements. His position was on his side when his son first saw him. He turned him on the back, and dashed water into his face to restore him, but with no effect.

*Examination 12 hours after death.*—Present, Drs. Loomis, G. N. Burwell, J. S. Trowbridge, and Wilcox.

The body presented large muscular development, with a thick layer of adipose substance under the external tegument.

*Head* first examined. On dividing the scalp, fluid blood flowed in great abundance. Nearly half a pint escaped on division of the scalp alone. On removing the calvarium, fluid blood escaped freely from the divided vessels connecting the dura-mater with the skull. Blood constantly exuded from the surface of the dura-mater, especially over the line of the longitudinal sinus. On detaching the dura mater the arachnoid was somewhat elevated by sub-arachnoid effusion of serum, slightly opaque, causing the surface of the brain to present the appearance of arachnitis, with fibrinous exudation beneath it. On dividing the arachnoid, however, serum flowed freely, and no fibrin was discoverable. The vessels of the pia mater were considerably, but not intensely, injected. The arachnoid appeared somewhat firmer than usual. On removing it, the surface of the brain presented a dull leaden color. On section of the cerebral substance, red points were thickly scattered over the incised surfaces. The lateral ventricles contained a small quantity of limpid effusion. The cerebrum was separated on a level with the tentorium, and removed. The head was elevated, but, nevertheless, bloody serum constantly escaped from below the tentorium, anterior to the pons varolii. On dividing the tentorium, and removing the cerebellum, a considerable quantity of sero-sanguinolent fluid was found at the base of the skull. On depressing the head, the same kind of fluid flowed freely from the spinal canal. It was not definitely ascertained whether the sanguinolent appearance was due to the admixture of blood from the divided vessels, in separating the cord, or whether it possessed this character before making the separation. The surface of the cerebellum exhibited considerable vascular injection. No softening of cerebral substance discovered. The vertebral arteries protruded from the divided surface of the medulla oblongata, and, on removing them, were found to be ossified. The carotids, as they enter the brain to form the circle of Willis, were more rigid than usual, but distinct ossification was not discoverable.

*Chest.*—Little or no flow of blood on division of the integuments. Lungs healthy, with moderate congestion at inferior portion. Pericardium contained very little serosity. The heart was manifestly enlarged, but not greatly so. Its transverse diameter was somewhat increased, giving the organ a more spherical appearance than natural. The left ventricle was opened before removing the organ. This cavity contained some fluid blood, but was not distended. A small coagulum (as large as an almond) was contained within it. The vessels were divided. The flow of blood was not great. The left ventricle and aorta were now slit open. The sigmoid valves were more rigid than usual, but in the valves themselves there was no ossific transformation. At the line of junction, however, of two of the valves, ossific matter was deposited, causing a fixed protrusion of the valves. The mitral valve presented no ossific transformation. The chordæ tendinæ were more rigid than usual, but no bony deposition. The left ventricle was of more than normal thickness, exceeding an inch, except at the left latero-posterior portion, where the walls were very thin, and had lost the muscular character, being converted into cartilaginous tissue. Right sigmoid valves healthy. Right side of heart not distended with blood. Stomach not distended. Colon considerably distended with flatus. Examination not extended to other organs.

*Remarks.*—That the immediate cause of death, in this case, existed within the cranium, will not be doubted. The patient died of apnoea produced by cerebral congestion and effusion. What degree of relative agency is to be attached to the effusion within the arachnoid cavity? It is difficult to answer this question with precision, but that the amount of relative agency was considerable, seems a rational supposition. It may be questioned, if congestion alone, however intense, would be likely to destroy life so suddenly—coma would have continued for some time before a fatal termination. The effusion of a considerable quantity of fluid into the arachnoid cavity, compressing the medulla oblongata, certainly affords a more rational explanation of the sudden death, than the congested state of the vessels alone.

The effusion was probably due to the congestion, being perhaps a mechanical transudation through the coats of the over-distended vessels. It is not to be overlooked, in these cases, that, to some extent, serous transudation may take place after death.

It is easier in this case to account for death, from the cerebral congestion and effusion combined, than to understand why so great determination of blood to the head should have occurred. The morbid appearances found in the heart do not appear to be sufficient to have caused a degree of obstruction adequate to such a result. The fact is the more surprising, inasmuch as it must have been almost instantaneous, there having been no symptoms of cerebral congestion, or other trouble, an hour, at farthest, before the death of the patient.

This case afforded a striking demonstration of the incorrectness of the idea, advocated by Dr. Kellie, and adopted by Clutterbuck, Abercrombie and others, that the brain, under all circumstances, contains a given amount of blood, which, for physical reasons, can never vary. This hypothesis has been abundantly disproved by the recent experiments of Dr. Burrows.

CASE 7.—*Sudden death in convulsions (child). Tuberculous deposit in lungs. Effusion into arachnoid cavity, and into lateral ventricles. Tubercular deposit on surface of cerebellum.*

The subject of this case was a child (girl) aged 4½ years. In the autumn of 1846 she had an attack of croup, the urgent symptoms being speedily relieved by an emetic of Turpeth. mineral. I discontinued visits after two days' attendance. Subsequently, however, she had two slight attacks, which were relieved by some domestic remedies, medical aid not being requested. Several weeks afterwards she had parotiditis.

About the 20th of January, 1847, I was requested to visit her, and found she had not been well since the attack of mumps. She had now febrile movement, especially at night, loss of appetite, and some cough. It was ascertained, about this time, that vision, in one eye, was lost. This discovery was casual, and it became a question whether it was an event of recent occurrence. As no difference in the appearance, or motions, of the eye was perceptible, the presumption was, that it had existed for some time, before it was discovered. Dr. Hamilton saw the patient in consultation with reference to the loss of vision, and concurred in the above opinion. She had had inflammation in the eye during the preceding summer, not severe, and no medical aid was requested. It was supposed that retinitis might have occurred at that time, which was followed by amaurosis.

She remained in a stationary condition for two or three weeks, when, at length, the febrile movement abated, her appetite improved, and she seemed decidedly improving. On the 4th of February she appeared to her friends to be decidedly better. I did not see her on that morning, visiting her only on alternate days. She was brighter than usual, had more appetite, and made an effort to go about the room. At about 3, P. M., after having slept a short time, she awoke, and shrieked, apparently with pain in head. Very soon her countenance assumed a vacant, staring expression, and she manifested delirium. I saw her half an hour afterward. She had then a staring look, pupils dilated, and the pupil of the sound eye most so. She had then some degree of vision, looking at her hands with a staring, surprised expression. She soon fell into convulsions, the muscles of the face, and of the upper and lower extremities, being affected. The right lower and the left upper extremity were affected. The convulsions continued, with occasional relaxations for a few moments, but never a complete interval, until 10½, P. M., when death occurred. I regret to find that the record of the case does not contain any reference to the functions of respiration or deglutition during the convulsions.

*Post mortem examination,* 36 *hours after death.* Present, Drs. G. N. Burwell, and J. G. House.

*Head.*—Considerable congestion of the superficial veins of the encephalon. Section of the cerebral substance presented numerous red points. No effusion apparent beneath the arachnoid, nor opacity of this membrane. *At the base of brain there existed, as estimated, from one and a half, to two ounces of slightly opaque serum.* Slight effusion into the lateral ventricles. At the decussation of the optic nerves there existed what appeared to be an abnormal collection of cellular tissue. On the upper surface of the cerebellum, a space about the size of a half dollar was studded with numerous white dots, presumed to be tuberculous deposits. No other pathological appearances.

*Chest.*—In the middle lobe of the lung in the right chest, there was found a solid collection of the size of an English walnut, consisting of crude tuberculous matter in irregularly shaped masses. A few drops of purulent looking fluid escaped on section of one of these masses; otherwise they were entirely crude. Miliary tubercles disseminated sparingly throughout the lung.

Other organs not examined.

*Remark.*—The supposition of sudden effusion into the arachnoid cavity, affords, in the opinion of the writer, the most rational explanation of the attack of convulsions and death in this case.

CASE 8.—*Patient, a child, aged six months. Sudden death several days after the operation of hare lip. Autopsy. Effusion into arachnoid cavity.*

I was invited by Dr. F. H. Hamilton, on the 12th August, 1848, to be present at the autopsy of a child, six months old, on whom, a few days previously, he had operated for hare lip. The child had had diarrhœa prior to the operation, which was relieved at the time the operation was made. It seemed to be doing well after the operation, until the evening previous to the day of its death. Dr. H. was then called in, and found slight febrile movement, and accelerated respiration, but did not deem the smptoms sufficient to require any active treatment. It slept well during the night, and in the morning the mother thought it better. Suddenly it became worse, (the precise symptoms I did not gather. It had no convulsions,) and before Dr. H. could reach the house, although living in the immediate neighborhood, death had taken place.

On examination of the brain, the superficial veins were moderately injected. No red points on section of the cerebral substance. Lateral ventricles empty. *From 1½ to 2 ounces of fluid at base of the skull.*

*Chest.*—Lungs perfectly healthy. Heart normal: foramen ovale closed.

*Abdomen.*—Several invaginations of small intestine, all readily restored, without evidences of inflammation.

CASE 9.—*Patient* 11 *years of age. Presenting physical evidences of incipient tuberculosis of lungs. After headache of five days' continuance, not deemed important, respirations became sixty-two in a minute, and irregular, the pulse being eighty and irregular. Double vision followed, and death in nine days after date of the accelerated respiration. Slight arachnitis over inferior surface of the cerebellum, and effusion within the arachnoid cavity, amounting to between three and four ounces. Slight effusion into lateral ventricles.*

The case, of the important features of which, in so far as the present subject is concerned, the above is a synopsis, was reported for this Journal, by the editor, in Vol, IV., page 744, (No. for May, 1849,) The attention of the reader is referred to the details of this case, in connection with the present subject. It is the most interesting that has fallen under the notice of the writer. I will take the liberty of quoting that portion of the remarks published in the report of the case, which relates to the cerebral effusion, and the circumstances upon which a diagnosis was based:—

"We may regard the sub-acute arachnitis, existing over a small, circumscribed space, situated just posteriorly to the medulla oblongata, as the pathological condition to which the result, and antecedent phenomena of this case are to be ascribed. The inflammation was so limited in extent, and of so little acuteness, that the early symptoms gave no intimations of the character of the disease, or the danger of the patient. The development of the symptoms noted on the 16th of March, five days after headache was first experienced, marked the occurrence of effusion, sufficient to disturb, by compression, the functions of the medulla. The combination of symptoms then exhibited, indicated, in the first place, serious encephalic trouble. These symptoms were, irregular and greatly accelerated respirations, in connection with diminished frequency of the pulse; coolness of the surface; continued pain in head, more marked in occiput; and the absence of any evidences of a new pulmonary affection. It is to be observed, that notwithstanding the respirations were so frequent that the patient appeared to be panting for breath, no sense of embarrassment, or dyspnœa was experienced. This denoted that the cerebral cause was so situated, as to impair the instinctive sense connected with the respiratory function. The fact that the phenomena of a grave cerebral affection, were almost exclusively manifested in the respiration, together with the absence of symptoms appertaining to inflammation, or other disease affecting the cerebrum, was sufficient to warrant the conclusion, that the disease was situated so as to involve the medulla oblongata, the direct relation of the latter to the respiratory function, being considered. Hence the diagnosis, in so far as the situation of the affection was concerned, the correctness of which was verified by the autopsy. The same amount of arachnitis, elsewhere situated, for example on one of the lateral surfaces of the cerebral hemispheres, would not have led to such a combination of symptoms—characterized by so disproportionate disturbance of the respiration; and situated, as it was, it was probably not sufficient to involve imminent danger, except from the effusion which occurred. Our attention has been directed, by several cases that have come under observation, to the occurrence of effusion at the base of the brain, and we are satisfied that it is a not unfrequent cause of death, occurring unexpectedly, and more or less suddenly, especially in children. Every observing practitioner is aware that disproportionate disturbance of the respiration, irrespective of pulmonary disease, is generally of bad omen, in whatever pathological connections it may be found. It is so, for the same reason that it was significant in this case, denoting encephalic trouble, involving the medulla oblongata, leading, in fatal cases, to asphyxia, by destroying the physiological sense presiding over the involuntary movements, necessary to the respiratory function."

CASE 10.—*Headache of several days' continuance, unattended by symptoms denoting encephalic inflammation, followed by paroxysms of stupor, bewilderment, and general distress, irregularity of pulse. Death. Congestion of brain, and effusion within cavity of the arachnoid.*

I was requested to visit Miss C., aged 20, on the 3d of Jan., 1858. She had generally enjoyed good health. During the last winter she was thought to have inflammation of the lungs, and was attended by a homœopathic practitioner. She had had no symptoms of pulmonary disease during the last summer and Autumn. Aspect denoted health. My advice was requested in consequence of headache, of which she had complained for several weeks, but more especially for several days previous to my visit. Her expression was dull and heavy. She seemed taciturn. Her friends afterward informed me that her temper had of late appeared changed. She was disinclined to see persons, or to converse, and was irritable. The face was somewhat flushed. No unusual throbbing of carotids, nor sensibility to light or sounds. Pain not acute or lancinating, but dull and constant. Extremeties not cold, but temperature normal. Pulse not accelerated. Menses regular. Bowels constipated. No nausea. Appetite tolerable. I directed laxative medicine, and desired to be informed if the headache was not relieved.

On the 4th, I was again called. Headache and heaviness were somewhat increased. Directed Croton oil, in pills, each pill containing half a drop, to be given hourly, until free cathartic operation.

On the 5th A. M. she reported better. Still considerable headache, and sense of heaviness, or weight in head, continued.

In the afternoon of the same day I was sent for in haste. She had appeared to the friends to have a fit. She seemed lethargic for a few moments, not replying to questions, and could not be aroused. This continued but for a few moments. When I reached the residence, she was sitting up—aspect dull; complained of pain in head, which was not severe. She continued, as before, taciturn, disinclined to describe her sensations, replying in short sentences. The treatment consisted of antimonial solution hourly, mustard foot-bath, and the application of cold water to the head.

On the 6th, reported better. Had passed a comfortable night. Antimony had vomited slightly. Pulse, as heretofore, not accelerated. Some appetite. Directed two leeches to head. Cold applications continued after the leeching. She set up a portion of the time, up to this date, and occasionally walked about the house. In the evening I was summoned in haste. She had had two or three paroxysms of bewilderment and distress. The paroxysms came on suddenly, and were characterized by exclamations of distress, saying "oh! dear, oh! dear." She apparently had little or no cognizance of persons around her; would not reply when addressed. She put her hands to her head at these times, and made attempts to get out of bed. These paroxysms lasted for four or five minutes. They continued to occur, with about half an hour's interval, and in the interval she was in a quiet, dozing state. She now begun to refer the pain to the nucha. She said she did not know how she felt. She was disinclined to converse. She was bled about ℥xvi, and spts. lav., comp., and tinct, assafœtida given after each paroxysm.

On the 7th, she seemed again comfortable. Pulse not accelerated. Skin normal. Tongue moist and furred. Complained of pain wholly in nucha. A blister to nucha was applied, and calomel directed, grs. v, every four hours, until free cathartic operation. She passed the day comfortably. Pain in nucha continued. Skin cool. Pulse scarcely, if at all, accelerated. Respiration normal. No cough. Percussion elicited clear resonance on both sides of chest. Sounds and impulse of heart normal.

8th.—Passed a tolerable night, but had some return of paroxysms similar to those which occurred on the 6th inst. Calomel had operated once, freely, complained of pain in nucha and back. Aspect improved—less dull and heavy. Said she felt better.

At evening nothing new. I directed at night, sulph. morphiæ gr. $\frac{1}{24}$, tart. ant. et pot, grs. $\frac{1}{12}$ every two hours. Complained this evening of pain in side. This evening, for the first time, the pulse became considerably accelerated, being 120.

9th.—Summoned early. She had, in the early part of the night, fallen into a lethargic state, and had so continued during the night. She could not be aroused. She lay part of the time with her eyes open, and close approximation of the finger did not occasion winking. This was her condition when I first saw her. She could not be aroused to reply to questions, but, on being repeatedly requested, she protruded the tongue fully. Respiration normal. Pulse not much accelerated, and well developed—*somewhat irregular*. The latter feature I had before noticed. She assumed a natural decubitus on the side, and occasionally put her hands to her head. *Fluids collected in the throat, showing that the faculty of deglutition, or the sense of the presence of fluids in the throat, was impaired.* Dr. White saw her, in consultation, on this morning. With considerable effort, she was, at length, aroused to swallow drink, to hold the cup in her hand, and to reply to questions. Six leeches were applied to the temples.

Ice cap to head. Ol. Tiglii, gtt. 1, hourly, until operation. At noon she was conscious. Replied to questions readily. Face presented a haggard expression. Leech bites were still bleeding. Said she did not know how she felt. Oil had not operated. At evening visited again with Dr. White. She was then in profound coma, with tracheal rattle, and hippocratic countenance. She had fallen into a state of complete unconsciousness, soon after my last visit, at noon. *Respiration not stertorous, nor did it present marked irregularity in rhythm. The pulse was well developed, and not much accelerated.* Death was evidently taking place by apnœa. Death occurred some hours afterward. The precise time of its occurrence is not stated in the record.

*Examination of the head, on the* 11*th inst.*—Present, Dr. White.

Calvarium and external surface of dura mater presented nothing abnormal, except that the latter was darker than usual. Slight escape of serosity, on dividing the dura mater.

Superficies of the brain, after the dura mater was removed, presented very considerable congestion, and moderate sub-arachnoid effusion. Cut surfaces of substance of brain presented dark points, and appreciable drops of blood. Plexus choroides congested. Lateral ventricles empty. *At base of brain, about an ounce of slightly opaque serous effusion was apparent, without elevating the body to allow of the flow of serum from the spinal canal.*

Arachnoid at the base of cerebellum appeared firmer than usual, and was slightly opaque. Consistence of brain normal.

Other organs not examined.

*Remarks.*—I was prepared to appreciate the dangerous character of the affection in this case, by having met with a case, many years ago, resembling it closely in the symptoms and post-mortem appearances. The latter case I have not included in this series, because, for the last two or three days before the death of the patient, I was not in attendance, and have no record of the symptoms immediately preceding the fatal termination. Such cases are certainly rare, and their gravity may be overlooked. They may be considered cases of hysteria, which would, of course, be a far less serious affection. So far as I know, Dr. Abercrombie's treatise on diseases of the brain, is the only work containing the histories of similar cases. Dr. Abercrombie entitles the affection "A dangerous modification of the disease, (meningitis,) attended only by increased vascularity." There are, however, as Dr. Watson remarks, insufficient grounds for regarding these as cases of meningitis. Increased vascularity, without the effusion of fibrin, denotes only congestion, not the state of inflammation. The fatal result was doubtless due to pathological conditions of the encephalon, and these conditions, so far as appreciable, were congestion, and effusion beneath the arachnoid, and into its cavity. Without attempting to define the exact relative influence of each of these conditions, it seems most rational to conclude that the *time* and *mode* of death was determined, chiefly, by the effusion into the arachnoid cavity.

We may suppose that up to the noon of the last day of the life of the patient, congestion alone, or with a small amount of effusion, existed, producing the tendency to stupor. But shortly after noon she fell, suddenly, into profound coma. Deglutition was lost: the *besoin de respirer* was also compromised, as shown by the accumulation of fluids in the trachea, although the respiratory movements did not exhibit, in this case, the usual disorder. The patient evidently died of asphyxia, the pulse being well developed, and not much accelerated, when she was evidently in the article of death. These circumstances appear to show the occurrence, in the progress of the disease, of some event impairing the functions of the medulla oblongata, in a degree incompatible with life. Had this event (arachnoid effusion) not occurred, the fatal termination would, to say the least, have been at a later period. The congestion alone, together with the sub-arachnoidean effusion, would not have destroyed life so soon, nor so distinctly by apnœa, the forces carrying on the circulation being, at the same time, so little affected.

The two following cases, having some bearing on the present subject, were reported by the writer, for the Illinois Med. and Surg. Journal, No. for November, 1844:—

"Case 1.—*Sudden death from (probable) sudden effusion at base of cranium.*

"By the politeness of Dr. Sands, attending physician to the alms-house of this county, I was invited to be present at an autopsical examination of the body of a woman, who had suddenly died, under the following circumstances:

"She had been committed as a vagrant, on the 27th inst., and was in a state of inebriety, when brought to the alms-house. On the 18th, she was dressed, and about the wards. Did not report herself as being ill; and no particular notice was taken of her condition. She was heard to say that she believed she should die if she could not have some whiskey. It was supposed by the attendants that she was suffering from the after effects of excessive indulgence in ardent spirits; but her general aspect did not indicate any severe ailment, nor did she apply for medical treatment.

"At about 2, P. M., on the 18th, while standing in one of the wards, she suddenly threw herself on a bed nigh at hand, and appeared to those in the room to have '*a fit.*' Neither the physician nor resident pupil were in the house. The keeper was immediately summoned, and arrived at the room in three or four minutes after the attack. He stated that she gave wo or three respirations after he entered the room, with long intervals between them, and ceased to breathe. He observed no convulsions.

"*Examination* 20 *hours after death.*—Body large, and well developed, and with considerable *embonpoint.* Age about 30. The face and neck, anteriorly, as well as posteriorly, presented deep lividity. This partially disappeared, after the thorax was opened, and the large vessels divided.

"As the objects of the examination were limited to the discovery of the immediate cause of death, it was proposed first to inspect the heart and other organs of the chest.

"In opening the pericardium, a sanguinolent fluid was observed to escape. It was estimated that about two ounces were contained within the pericardium. A slit, about half an inch in length, was observed in the right ventricle, corresponding to the incision through the pericardium. It was undoubtedly made with the scalpel of the operator, although care was taken to guard against this accident. The walls of the right ventricle were morbidly thin, but not softened. The slit had the appearance of having been made with a sharp instrument. There was no ulceration or softening about the aperture, nor any indication of endo-carditis. The general dimensions of the heart were not measured, but estimated to be normal; valves unaffected; no coagula within the cavities. The only morbid appearance was the attenuation of the walls of the right ventricle. This ventricle was probably distended, and being in close apposition with the pericardium, received the point of the scalpel, giving rise to the sanguineous effusion which exuded after dividing the pericardium.

"Lungs engorged throughout; excessively at their inferior portions, and considerably at the superior and anterior portions; otherwise no morbid appearances.

"The liver was enormously hypertrophied. It extended quite into the left hypochondrium, and was adherent to the diaphragm over a considerable extent of surface, both in the right and left hypochondrium. Its color was light yellow. The hypertrophy of the yellow portion manifestly predominated. It weighed seven pounds and two ounces.

"The stomach was larger than usual; not distended; its internal surface not examined. External appearance of intestines healthy.

"The object of the autopsy not being attained, the head was next opened.

"The dura mater adhered with great firmness, so that in the separation some injury was done to the brain, occasioning a flow of serosity, the amount of which could not be well estimated. There existed manifestly considerable effusion at the base of the cranium, within the cavity of the aarchnoid. It was sanguinolent, but this may have been owing to the rupture of blood vessels in the removal. Bloody fluid flowed from the spinal canal. The brain presented moderate congestion. It is to be borne in mind, with reference to this, that the chest was *first* opened, and the large vessels divided, which would tend to diminish the quantity of blood within the cranium. Aside from the congestion, appearance of the brain and membranes healthy. Slight effusion into ventricles.

"I should have remarked that fluid blood flowed copiously from the vena cava, when divided.

"*Remarks.*—The most rational explanation of the sudden death in this case, would appear to be—Congestion, and sudden effusion at the base of the brain, compressing the medulla oblongata, causing a cessation of respiration, and fatal asphyxia. The congestion and effusion were probably induced by excessive inebriety, and perhaps, in some degree, promoted by obstruction to the circulation, resulting from the diminished contractile force of the right ventricle, in consequence of its attenuated walls."

"CASE 2.—*Sudden Death, with large Coagula in the Heart, and effusion at base of Cranium.*

"By a singular coincidence, I was invited, on the same day, to be present at the autopsical examination of a second case, in which death took place as suddenly as in the other instance. The circumstances were briefly as follows:

"A boatman, aged about 35, came into the office of Drs. Wilcox and Rogers, of this city; was observed to stagger as he entered; seated himself in a chair, and uttered the word 'Doctor.'

"It was observed by Drs. Wilcox and Rogers, who were both present, that his eyes had a fixed, staring impression, and that the pupils rapidly dilated. Dr. Wilcox, perceiving that he was about to fall, seized him by the arm, and allowed him to slide from the chair to the floor, extended him upon his back, and dashed cold water in his face, supposing it might be syncope. He gasped two or three times afterwards, his limbs were spasmodically extended twice, and he expired, as estimated, about two minutes after entering the office.

"It was ascertained, by evidence at the coroner's inquest, held immediately, that he was of intemperate habits, had suffered much from intermittent fever, and, for some days past, had had diarrhœa. A person who had seen and conversed with him a short time previous, on the same day, stated that his respiration was hurried and labored; that he complained of distress in the stomach, and that his general aspect was exceedingly bad.

"*Examination two hours after death.*—Body considerably emaciated, face pallid, and features contracted. Presented the appearance of a subject dead after lingering disease.

"Head was first examined.

"Adhesion of dura mater of ordinary firmness; meningeal veins moderately congested; sero-sanguinolent fluid escaped from within the dura mater at the posterior portion of head. Care was taken to elevate the head while the brain was being removed, and, as estimated, more than two ounces of sero-sanguinolent fluid was found at the base of the skull. Section of brain presented more red points than usual, otherwise no morbid appearances.

"*Chest.*—About an ounce of transparent serum in pericardium. Right auricle contained a firm yellow coagulum of lymph, about the size of a small hen's egg. It seemed quite to fill the cavity. A prolongation extended through the auriculo-ventricular orifice. It was firmly interwoven with the musculi pectinati, so as to be with difficulty detached.

"A coagulum having the same appearance, but of less size, existed in the right ventricle, firmly intertwined with the chordæ tendinæ. From this coagulum, a prolongation of about half the caliber of the pulmonary artery, extended upward about half an inch beyond the sigmoid valves. The endo-cardial membrane, both of the right auricle and ventricle was remarkably white, and presented no evidence of disease; the left auricle and ventricle nothing abnormal. Dimensions of heart not measured, but estimated to be below the normal size, if any variation existed. Blood flowed copiously from the cavæ when divided, which was fluid when it first escaped, but in a short time formed loose, dark coagula.

"Lungs deeply congested, otherwise healthy.

"Liver greatly enlarged; of dark red color; deeply congested with fluid blood. Stomach and other viscera not inspected. The objects of this examination were limited to the discovery of the immediate cause of death."

The question may arise, respecting the second case, whether the immediate cause of death was not the presence of coagula in the heart, which had formed during life. That they had formed during life is altogether probable, for the examination was made two hours after death, and the blood in the large vessels was still fluid, coagulating, in part, after being removed from the vessels. Doubtless this contributed to the fatal result, and was the first link in the chain of cases immediately concerned therein. It seems, however, probable, that the cause standing most directly to the sudden death, was the effusion into the cavity of the arachnoid. This effusion, probably, occurred in consequence of cerebral congestion, induced by obstruction to the circulation through the right auricle produced by the large coagula in that cavity.

The foregoing twelve cases are given with a view to inviting the attention of the profession to effusion into the arachnoid cavity, as a pathological event, in a great measure overlooked by medical writers, and the importance of which does not appear to be sufficiently appreciated. They are submitted, also, as a slight contribution toward materials for comparison with reference to a generalization of laws regulating the occurrence of this event, and a determination of the symptoms which denote its occurrence. With reference to the latter point, the writer is well aware that several of the histories are deficient in details which it were desirable should have been recorded. Most of the cases occurred several years ago, when the importance of these details was not so apparent to the mind of the writer as now. This is the only apology he has to offer for omissions which he greatly regrets.

The length to which this communication is already extended, precludes the discussion of several points which had been reserved for concluding remarks. For the sake of brevity, the following propositions are submitted, embodying considerations which appear to the writer to merit investigation. They are not presented as established truths, but as invested by facts with probability, and to be proved, or disproved, by reference to a larger collection of facts. With regard to some of the propositions, a few comments will be indulged:

1. Effusion into the arachnoid cavity, at the base of the brain, is an occurrence liable to take place as an intercurrent event in the course of various affections. It generally involves cerebral congestion in its immediate causation. Cerebral congestion, leading to this result, may be induced under a variety of circumstances, physical and vital. We have no means of predicting the occurrence of effusion into the arachnoid cavity. As respects our ability to anticipate it, it is to be regarded as a species of accident.

2. Effusion into the arachnoid cavity is not an infrequent cause of sudden death. Sudden deaths, occurring without being preceded by obvious disease, and fatal terminations occurring unexpectedly in the progress of various diseases, may be due to this cause. This may be considered a variety of serous apoplexy.

The propriety of regarding serous apoplexy as a verity, has been discredited by some modern writers. Others, however, regard the effusion of serum as constituting one of the pathological conditions upon which apoplexy is dependent, but they refer exclusively to effusion within the ventricles. This is the case with the late Dr. Gòlis, of Vienna, who has treated of this subject under the head of *Wasser Schlag*, or water stroke. Dr. Abercombie, in his sterling, classical work on diseases of the brain, gives histories of several cases in which, among other pathological appearances, the existence of effusion as the base of the brain is mentioned, but not referred to as a matter of importance. So, this fact may be found noted in various reports of autopsies, by different authors, but generally without attention being directed to it as a point meriting much consideration. Dr. Abercrombie entertained the belief, that when serous effusion was discovered in the encephalon, after an apopleptic seizure, the latter was due to the state of the vessels preceding and occasioning the effusion, rather than to the compression produced by the effusion. In this view he has been followed by most other writers. It may fairly be doubted if this view can be sustained.

Dr. A., also, as is well known, details a case in which, after apoplexy, proving fatal, no morbid appearance of the brain was discoverable; and another case in which only moderate engorgement of the vessels was found. To explain such cases, (which Dr. A. entitles cases of "Simple Apoplexy,") it is supposed that sufficient congestion to produce the apoplectic attack may have existed during life, but, owing to physical causes, the engorgment of the vessels diminished, or disappeared after death.

Is it not more rational to suppose, that in these cases, effusion at the base of the brain existed, which may have been overlooked, inasmuch as so little importance has generally been attached to it? Without presuming to doubt the ability of Dr. Abercrombie, as an observer, may it not be considered possible that, with his views relative to the pathological importance of cerebral effusions, his attention may not have been directed to the presence of a fluid in the arachnoid cavity in his cases of simple apoplexy? The views, with respect to effusion, entertained by Dr. A., perhaps afford some explation of the fact that this subject has failed to receive adequate attention from others.

3. Death from effusion into the cavity of the arachnoid is produced by apnœa, or asphyxia, induced in consequence of mechanical compression of the medulla oblongata, suspending the *besoin de respirer*, and the muscular acts carrying on the respiration.

4. The circumstances involved in the diagnosis of this accident are as follows:—Somnolency, or coma, without paralysis, not preceded by the symptoms of meningitis; disordered respiration; deglutition lost or impaired; intellect not disordered, if the patient is susceptible of being roused; forces carrying on the circulation not affected in proportion to the disturbance of the respiration and deglutition; the *sudden* development of grave cerebral trouble, and rapid tendency to a fatal result by apnœa

The case, among those which have been reported in this communication, which presents the best illustration of the symptoms due to effusion into the arachnoid exclusively, is No. 9, (the case reported in full in the No. of this Journal for May, 1849.) Usually, the effusion being connected with considerable congestion, and perhaps other cerebral disease, the symptoms of all are commingled, and the fact of the effusion is to be predicated upon the characteristic symptoms being in disproportionate excess.

5. The gravity of the symptoms, the danger of the patient, and the suddenness of death, will be commensurate with the *rapidity* with which the effusion takes place, as well as the *amount* of the effusion. This is rationally and analogically probable. It follows from this, that the quantity of fluid bears no fixed relation to the symptoms and danger, and that, in post mortem examinations, the pathological importance to be attached to the existence of effusion, does, by no means, relate exclusively to its amount.

6. Insolation, or *coup de soleil*, when it proves fatal, involves effusion into the cavity of the arachnoid.

This is submitted as an inquiry, the writer having had no practical acquaintance with this affection. The phenomena seem best explicable on the supposition of the occurrence of effusion at the base of the brain. It has been sufficiently established by the observations of Mr. Russell, British Army, Dr. Gerhard, of Philadelphia, and Dr. Dowler, of New Orleans, as well as others, that the mode of death in insolation is by asphyxia, and that the evidences of marked cerebral congestion are not necessarily present. In one of three dissections given by Mr. Russell, (see lectures by Dr. Graves,) it is stated that a small quantity of serum was effused at the base of the brain, no importance appearing to be attached to it by that writer. May not the existence of this pathological event have been overlooked in such cases?

In an able communication, published in the New York Medical Gazette, No. for October, 1841, [vol. 1, p. 209,] Dr. Bennet Dowler, of New Orleans, gives a description of this affection, which appears to the writer to establish the truth of this proposition, in so far as it may be established by the circumstances appertaining to the symptomatology of the affection. Dr. D. offers several considerations to disprove the hypothesis that the affection is apoplectic, but none of them are applicable on the supposition of effusion into the arachnoid sac. In describing the symptoms, he says:— "Almost always the breathing stops *suddenly*, by a strangling fit;" and, again, "from the commencement of the disease the power of swallowing is totally gone, in almost every case." The lungs are always found greatly congested, and, hence, this author entitles the affection *solar asphyxia;* but he remarks, "whether the pulmonary congestion be the primary or secondary condition of insolation, I will not say." His attention does not appear to have been directed to the arachnoid cavity. His account of pathological appearances contains no reference to this part. The characteristic symptoms given in this interesting paper by Dr. D, would go to show, (agreeably to the views that have been presented,) that the asphyxia is a secondary condition, dependent on compression of that portion of the central nervous system which presides, *par excellence*, over the respiratory function.

7. The presence of a fluid in the arachnoid cavity, sufficient in amount, or effused with sufficient rapidity, to produce grave symptoms, arising from compression of the medulla oblongata, will almost necessarily prove fatal, and it is doubtful if much is to be expected from therapeutical measures.

The last postulate of this proposition is based on the fact that the cause of death operates mechanically, and will, probably, if sufficient to occasion serious trouble, destroy life too speedily for therapeutical measures to prove efficient.

The indications, in cases in which a diagnosis is made, for medical treatment, is, plainly, to endeavor to procure, as speedily as possible, the absorption of the effused fluid.

The measures adapted to this object do not require to be pointed out.

BUFFALO, March 9th, 1850.

